# Lacunarity Analysis: A Promising Method for the Automated Assessment of Melanocytic Naevi and Melanoma

**DOI:** 10.1371/journal.pone.0007449

**Published:** 2009-10-13

**Authors:** Stephen Gilmore, Rainer Hofmann-Wellenhof, Jim Muir, H. Peter Soyer

**Affiliations:** 1 Dermatology Research Centre, The University of Queensland, School of Medicine, Princess Alexandra Hospital, Brisbane, Australia; 2 Department of Dermatology, Medical University of Graz, Graz, Austria; Tufts University, United States of America

## Abstract

The early diagnosis of melanoma is critical to achieving reduced mortality and increased survival. Although clinical examination is currently the method of choice for melanocytic lesion assessment, there is a growing interest among clinicians regarding the potential diagnostic utility of computerised image analysis. Recognising that there exist significant shortcomings in currently available algorithms, we are motivated to investigate the utility of lacunarity, a simple statistical measure previously used in geology and other fields for the analysis of fractal and multi-scaled images, in the automated assessment of melanocytic naevi and melanoma. Digitised dermoscopic images of 111 benign melanocytic naevi, 99 dysplastic naevi and 102 melanomas were obtained over the period 2003 to 2008, and subject to lacunarity analysis. We found the lacunarity algorithm could accurately distinguish melanoma from benign melanocytic naevi or non-melanoma without introducing many of the limitations associated with other previously reported diagnostic algorithms. Lacunarity analysis suggests an ordering of irregularity in melanocytic lesions, and we suggest the clinical application of this ordering may have utility in the naked-eye dermoscopic diagnosis of early melanoma.

## Introduction

With an incidence rate increasing over the last few decades, melanoma has become a major public health concern in many Western countries [1]. Although life expectancy in patients with advanced disease has not significantly improved during this time, the prognosis in patients with early and localised disease remains favourable. Hence the early diagnosis of melanoma is critical to achieving reduced mortality and increased survival [2]. Early diagnosis is facilitated by primary or specialist medical skin surveillance. For high-risk groups, such as those with multiple atypical or dysplastic naevi, photographic records enhance detection. Evidence suggests that dermoscopy – an *in-vivo* method capable of revealing sub-surface structures and improving colour resolution – can improve diagnostic accuracy in trained users [3], [4]. However, there exist limitations to the clinical assessment of melanocytic lesions. Poorly trained clinicians do not perform as well in melanocytic lesion diagnosis as their well-trained counterparts, and subjectivity, even among experts, is commonplace in dermoscopic pattern analysis [5]. The computerised analysis of melanocytic lesions is an endeavour that attempts to address these latter two problems [6]. Discriminatory software aims to help the poorly trained clinician achieve higher sensitivity and specificity in diagnosis, and the use of computer algorithms removes subjectivity in dermoscopy analysis. Computerised analysis of pictorial data can also be viewed as a third *in-vivo* window of critical analysis, complementing naked eye and dermoscopic examination. It is possible software can recognise features – in the digital representation and analysis of colour intensity throughout a lesion – when the eye cannot, and therefore improve the diagnostic accuracy of even well trained and experienced clinicians.

Lacunarity, a measure first introduced by Mandelbrot [7], and subsequently described by others [8]–[10], was initially used to characterise a property of fractals. However, lacunarity analysis can be applied to objects that are not self-similar [10]. Various investigators have taken advantage of this non-restrictive property by applying lacunarity analysis to imagery in a number of diverse fields including geology [11], ecology [12], radiology [13] and dermatology [14]. Lacunarity is a measure of translational invariance of an object [11], and quantifies aspects of patterns that exhibit scale-dependent changes in structure. Dermoscopic images of melanocytic lesions exhibit rich multi-scaled and multi-textured structures that we hypothesise are directly amenable to lacunarity analysis. Clumping of colour intensity at one or more length scales is associated with a violation of translational invariance and high lacunarity values, while colour or texture homogeneity is equivalent to translational invariance and low lacunarity values. Lacunarity values can be expected to correlate with, for example, heterogeneity of red, entropy, main axis asymmetry, border irregularity, and contrast – all key parameters known to be of significant discriminatory value in the automated differentiation of benign and dysplastic naevi from melanoma [15]–[17]. Indeed, lacunarity calculations have recently been implemented in the assessment of melanocytic lesions. Lacunarity was shown to exhibit higher values in melanoma compared with non-melanoma [18], and it has been proposed that lacunarity may have diagnostic utility as an independent parameter in melanoma diagnosis if combined with mean diameter and range of blue [14].

We perform our analysis as follows. The position of a polarised dermoscopic image of a melanocytic lesion is identified manually by locating the four points of the lesion that correspond to its upper and lower, and left and right extremities. The image is then cropped at these four reference points and lacunarity analysis is applied to the resultant standardised image. Our lacunarity calculation is a measure of the variation in either normalised 8-bit red, green or blue (RGB) colour intensity across the whole image. An 8-bit RGB image exhibits 2^8^ or 256 grades of color intensity in red, green and blue. Normalised intensities are rescaled to values between 0 and 1. A pixel is chosen at random within the image at location *m_i,j_*. Colour intensity is then summed for a range of box sizes *x^2^* centred at *m* with odd-valued edge sizes ranging from *x* = 3 to *x* = *x*_max_ pixels wide. For example, if *x_max_* = 7 colour intensity would be summed over boxes centred at *m* with sizes 9, 25 and 49 pixels. This procedure is then repeated for *y* randomly chosen *m_ij_*, and the *y* values with respect to a given box size are used to calculate lacunarity for that box size (see Methods). For the example above this would yield a lacunarity value associated with box sizes 9, 25 and 49 – the lacunarity vector. The maximum box size used (*x_max_*) will determine the length of the lacunarity vector, while the number of random centres (*y*) used is the centre count. The mean value of the lacunarity vector is then taken as the singular lacunarity measure for the image. Plotting the logarithm of the lacunarity vector values versus the logarithm of the corresponding box size yields a *lacunarity plot* – the slope of the line is a measure of the objects' fractal dimension while the correlation between the points and the line of best fit is a measure of the self-similarity, or “fractal-like” nature of the image [11].

There are significant differences between our methods and those reported above [18]. First, we minimise the problems associated with boundary recognition algorithms by using a minimal segmentation procedure. Second, we apply our image analysis to dermoscopic images rather than native images. Third, we measure 8-bit normalised red, green or blue colour intensity rather than either greyscale intensity or binary threshold red, green or blue colour intensity. Fourth, we measure lacunarity for different box sizes, generating a lacunarity vector for each image at one of three maximal box sizes. This methodology allows us to fully explore the capabilities of lacunarity in capturing geometric information about the distribution of colour intensity within an image.

Here we present the first comprehensive analysis of the utility of lacunarity in melanocytic lesion assessment. We show that this measure can discriminate melanoma from non-melanoma with a sensitivity and specificity comparable to previously reported diagnostic algorithms, and without many of the limitations of the latter. We find that the lacunarity measure suggests a natural ordering of irregularity in melanocytic lesions, and show how lacunarity analysis can reveal additional information regarding their geometric structure.

## Methods

### Image acquisition and pre-processing

Three hundred and twelve dermoscopic images of melanocytic lesions were obtained from the Department of Dermatology at the Medical University of Graz in Austria. All images were obtained from Caucasian patients and corresponded to one image per patient. No other demographic details were available. Digitised photographs were taken over the period 2003 to 2008. Polarised dermoscopic images of all lesions were obtained using a DermLite FOTO lens (3Gen LLC, Dana Point, California, USA) coupled to a digital camera (Nikon CoolPix 4500; Nikon Corporation, Tokyo, Japan) without flash using the camera's auto setting. Patient consent was obtained for the use of all images for research purposes, and all dermoscopic images shown are reproduced with permission.

Of the 312 lesions, 111 were considered benign by an expert dermatologist (RHW) using standard dermoscopic diagnostic criteria and were not excised. These lesions were used in another study [19]. Although it is possible some of these benign lesions were given an incorrect diagnosis, we expect the false negative rate to be very low, probably negligible, given that the diagnosis was made by an expert dermatologist with over 15 years experience in dermoscopy. Of the remaining 201 lesions, all were excised and examined microscopically by expert dermatopathologists using standard histopathologic diagnostic criteria. Of these excised lesions, 102 lesions were diagnosed as melanoma and 99 lesions were diagnosed as dysplastic.

Images were obtained as large JPEG files by one of us (SG) and were subsequently processed in two steps prior to analysis. First, the images were cropped to a rectangle or square such that the boundary of the lesion was adjacent to all four edges of the image (a very small number of the pre-processed JPEG's were of lesions that completely filled the frame). Second, each image was then compressed to a size where the shortest axis was 120 pixels wide. Three lesions were excluded following cropping, as their native resolution was less than 120 pixels along the shortest axis. Otherwise, all images were included and all artefacts, where present, were left untouched. Finally, to preserve differential information between images we did not perform histogram equalisation or brightness normalisation. Histogram equalisation removes contrast differences between lesions, while brightness normalisation removes differences in color intensity between lesions. These transformations have undesirable consequences for pigmented lesion diagnosis. For example, uniform benign lesions with subtle contrast and diminished red intensity may transform to lesions with high contrast and high red intensity – and thus be indistinguishable from unprocessed images of melanoma.

### The lacunarity algorithm

Lacunarity is defined as the non-dimensional ratio of the second and first moments of mass distribution. Here we equate mass with pixel RGB intensity, hence we are implementing lacunarity as a measure of the distribution of color intensity over an image. The first moment is equivalent to the mean 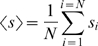



where *s_i_* is a mass element of edge size *x* centred at position *i* of *N* boxes covering the object. The second moment is equivalent to the sum of two components: the variance, given by 
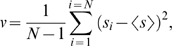



and the mean squared, denoted 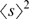, giving the lacunarity (*L*)
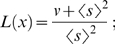
where *L(x)*, for the two-dimensional case, is a function of box size *x^2^*. In general, boxes are generated by a ‘gliding box’ procedure where a subset *N* of the total number of discretised elements (pixels) comprising the object are used as centres [11]; here we select centres at random.

For most purposes reported in the literature the maximal box size is one-half of the length of the smallest axis of the image; here we use three maximal box size diameters – one-eighth, one-half and one-quarter of the length of the smallest axis of the image, hence we performed three maximal box-size lacunarity calculations for each image.

The lacunarity algorithm assesses the red, green and blue components of the image separately. For each image the position of 1000 random pixels were generated consecutively by a random number generator and assigned as box centres. The first and second moments ranging from the smallest box-size (9 pixels) to the largest (either 225, 841 or 3481 pixels) were calculated about the centres as they were generated. This procedure generated a lacunarity vector for each image in the red, green and blue components. Depending on maximal box size, these vectors contained either 7, 14 or 29 elements. Images were thus assigned nine lacunarity values according to the mean of their red, green and blue vector values for each of three maximal box-sizes.

### Data analysis

Sensitivity and specificity ROC curves were generated automatically for each colour and maximal box size with a window range of 0.0025.

To determine whether there were significant differences between diagnostic groups, and since raw lacunarity values were skewed and could not be normalised by a suitable transformation, lacunarity values were compared using a three way non-parametric test (Kruskal-Wallis) and a two-way non-parametric test (Wilcoxon Rank Sum).

To assess whether there were any differences between diagnostic groups regarding their fractal dimension and degree of self-similarity (Pearson's correlation coefficient) non-parametric tests were used (Kruskal-Wallis and Wilcoxon Rank Sum) since these data were skewed and could not be transformed to normality.

To assess the correlation between the clinical irregularity scores and lacunarity values, Spearman's rank correlation was calculated using an index of 1 to 6 for clinical irregularity scores, and lacunarity values in the red spectrum at intermediate box size for each image. Finally, to assess the inter-observer agreement in clinical irregularity scores, a linear-weighted kappa statistic was evaluated.

## Results

### Validation of algorithm settings

**Figure 1:**
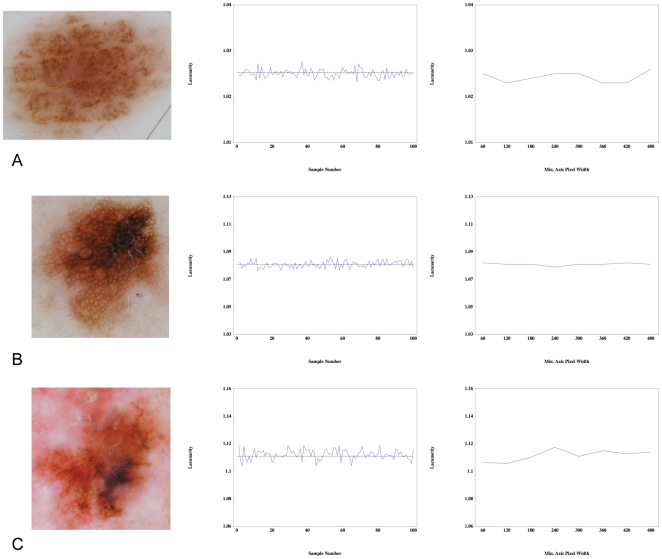
Lacunarity values as a function of box count and image resolution. Lacunarity values for the benign naevus (A), dysplastic naevus (B) and melanoma (C) of 100 repeated calculations where the box-counts were 1000 for each (column 2) and as a function of image resolution (column 3). For all plots in column 2 the points cluster about the true value (drawn as a horizontal line) obtained by exhaustive sampling (40,000 box-counts). Note that all data points shown in column 3 lay within the error range shown in column 2. The three native resolution JPEGs, with a minimum axis width of 480 pixels, are shown as the right-most data point of the plots shown in column 3. The lacunarity calculation at this resolution required over 12 hours of CPU time to complete.

The two important algorithmic settings that have a direct bearing on computational time in calculating lacunarity with respect to a given image are the centre count and the image size (see Methods). The values chosen for the centre count and minimum image axis size, 1000 and 120 pixels respectively, were found, after multiple test simulations, to be a reasonable compromise between accuracy, reproducibility and speed of computation. To invesigate accuracy and reproducibility, consider the melanocytic lesions and their associated lacunarity values as shown in Fig. 1. Here we have taken one lesion from each diagnostic group and repeated the lacunarity calculation 100 times. Compared with the values found by an exhaustive centre count of 40,000 – the true value – no values calculated with a centre count of 1000 were found to be in error by more than 0.008, a value two orders of magnitude smaller than the range of lacunarity values obtained from all images in this analysis. To investigate reproducibility, we performed a one-way ANOVA (analysis of variance) on the repeated lacunarity values from each diagnostic group. The within-group variation only accounted for 0.3% of the total (within and between-group) variation, thus demonstrating the reproducibility of the method. Finally, although native resolution analysis is prohibitively time-consuming since increases in image size were found to be associated with exponential increases in CPU time, all calculated lacunarity values, as a function of image size, and therefore CPU time, do not vary by more than 0.011 (Fig. 1). The compromise in accuracy with removal of image detail is therefore small and within the range of statistical uncertainty given by the centre count result.

### Algorithm diagnostic performance

**Figure 2:**
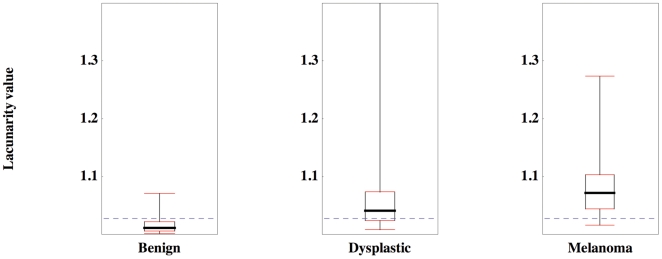
Box-plots of lacunarity values for the red spectrum at intermediate maximal box size with respect to diagnostic groups. Shown are medians, quartiles and 95% quantiles. The dashed line represents the optimal lacunarity value (*L* = 1.0275) that discriminates between melanoma and non-melanoma. Note the presence of outliers in the dysplastic naevi group.

The diagnostic performance of the algorithm was assessed with the centre count set to 1000 and with a minimal image axis width of 120 pixels. We evaluated lacunarity for all images in the red, green and blue spectra, and for three different maximal box sizes. We then sought to determine whether lacunarity values differ between the following pairs: dysplastic and benign naevi, melanoma and benign naevi, melanoma and dysplastic naevi, and melanoma and non-melanoma, and if so, which colour and maximal box size combination is the best discriminator. Receiver operated characteristic (ROC) curves were generated for all colours for all three maximal box sizes to determine whether lacunarity can distinguish between the diagnostic pairs above. We found that lacunarity analysis of images at the smaller and intermediate maximal box sizes was superior to image analysis at the larger box size, independent of colour, while the red spectrum was clearly superior to either green or blue, independent of maximal box size. Lacunarity values for the red spectrum at intermediate maximal box size for each diagnostic group are shown as box-plots in Fig. 2.

**Figure 3:**
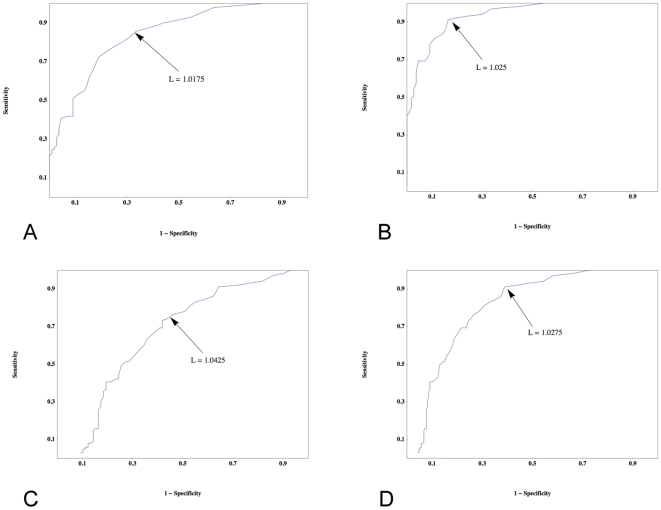
Receiver Operated Characteristic (ROC) curves. ROC curves are shown for the lacunarity measure of the red spectrum at maximum box-size equal to one-quarter of the shortest axis of the image. The following pairs are displayed: dysplastic v benign (A), melanoma v benign (B), melanoma v dysplastic (C), and melanoma v non-melanoma (D). Lacunarity can distinguish melanoma from benign naevi (B) with a SE of 92 and a SP of 81, however it does less well in distinguishing melanoma from dysplastic naevi (C), with an optimal SE of 76 and a SP of 54. A SE of 91 and SP of 61 distinguish melanoma from non-melanoma (D) with a discriminatory lacunarity value of 1.0275.

To determine optimal sensitivities and specificities from these ROC data, we used the following procedure: we took the 5 points where the sum of sensitivity and specificity was largest, and among those 5 points, chose the single point with the highest sensitivity, with the constraint that the specificity must be greater than 50. ROC curves for the red spectrum at intermediate maximal box size are shown in Fig. 3, with the optimal points highlighted. Optimal sensitivity and specificity results for different maximal box sizes in the red spectrum are presented in Table 1, while Table 2 shows the *T* and *z* test statistics for diagnostic group differences (including the Kruskal-Wallis test for three-way differences) in lacunarity as a function of colour for the intermediate maximal box size.

**Table 1:** Key lacunarity sensitivities (*Sens.*), specificities (*Spec.*) and 95% confidence intervals for melanocytic lesion diagnostic group discrimination.

	Max. box size*					
	*Smallest*		*Intermediate*		*Largest*	
	Sens.	Spec.	Sens.	Spec.	Sens.	Spec.
*Dys v Ben*	87 (78–92)	65 (56–74)	86 (76–91)	66 (56–74)	86 (76–91)	55 (44–64)
*Mel v Ben*	90 (82–95)	85 (76–90)	92 (85–96)	81 (72–87)	95 (88–98)	54 (44–63)
*Mel v Dys*	73 (63–81)	64 (54–73)	76 (66–84)	54 (44–64)	65 (55–74)	52 (42–62)
*Mel v Non-Mel*	88 (80–93)	66 (60–72)	91 (83–95)	61 (54–67)	84 (75–90)	55 (48–61)

*Ben*: Benign.

*Dys*: Dysplastic.

*Mel*: Melanoma.

*Non-Mel*: Non-Melanoma.

* Calculations are for the red spectrum.

**Table 2:** Tests for differences in means between diagnostic groups with respect to lacunarity (red, green and blue spectrum).

Inequalities	Test	*T* and *z* scores		
		Red	Green	Blue
*Ben v Dys v Mel*	KW (*T*)	141.9 *p* = 0.0000	21.4 *p* = 0.0000	18.0 *p* = 0.0000
*Dys > Ben*	WRS (*z*)	8.6 *p* = 0.0000	1.9 *p* = 0.0570	−2.1 *p* = 0.0360
*Mel > Ben*	WRS (*z*)	11.0 *p* = 0.0000	4.5 *p* = 0.0000	2.4 *p* = 0.0160
*Mel > Dys*	WRS (*z*)	3.9 *p* = 0.0001	2.8 *p* = 0.0050	4.1 *p* = 0.0000
*Mel > Non-Mel*	WRS (*z*)	8.8 *p* = 0.0000	4.3 *p* = 0.0000	3.7 *p* = 0.0001

KW: Kruskal-Wallis.

WRS: Wilcoxon rank sum.

*Ben*: Benign.

*Dys*: Dysplastic.

*Mel*: Melanoma.

*Non-Mel*: Non-Melanoma.

All calculations are for intermediate maximal box size.

Positive *z* values reflect significance for the diagnostic group inequalities as shown. *p*-values are given to 4 significant digits.

The results presented in Table 1 for the intermediate maximal box size include a lacunarity value (*L*) that can be potentially used as discriminator between diagnostic categories. For example, in our cohort of images, 91% of melanomas were associated with lacunarity values greater than 1.0275, while 61% of non-melanomas were associated with lacunarity values less than 1.0275. We then asked whether our lacunarity values possess any utility as a three-way diagnostic discriminator. Using *L* = 1.0175 (Table 1) to distinguish dysplastic naevi from benign naevi, and *L* = 1.0425 (Table 1) to distinguish dysplastic naevi from melanoma, (hence melanocytic lesions associated with lacunarity values between 1.0175 and 1.0425 would be classified as dysplastic) we find a diagnostic accuracy, when applied to all our images, of 0.66, 0.40 and 0.76 for benign naevi, dysplastic naevi, and melanoma respectively. These values are derived from the diagonal entries of the associated three-way matrix of confusion (Table 3).

**Table 3:** Matrix of confusion* for melanocytic lesion diagnosis.

	*Benign*	*Dysplastic*	*Melanoma*	Total
*Benign*	73	14	3	**90**
*Dysplastic*	27	39	21	**87**
*Melanoma*	10	45	77	**132**
**Total**	**110**	**98**	**101**	**309**

* This matrix is interpreted as follows: for 110 benign lesions, found in the first *column*, the algorithm diagnosed 73 as benign, 27 as dysplastic and 10 as melanoma. The first *row* shows the number of lesions diagnosed as benign, with respect to their true diagnosis. The remaining columns and rows, representing dysplastic naevi and melanoma, are interpreted similarly.

### Lesion irregularity and the structure of melanocytic lesions

We next examined the diagnostic groups with respect to their fractal dimension and degree of self-similarity (Table 4). Here we analysed data for the red spectrum at intermediate maximal box size. We found that there were significant differences in the fractal dimension between groups; the largest difference was found between benign naevi and melanoma where the fractal dimension was closer to 2 in the former. We then assessed the degree of self-similarity of images from different diagnostic groups by determining how well the points of the lacunarity plot fit the line of best fit; this is given by Pearson's correlation coefficient (*R^2^*). Although the Kruskal-Wallis test yielded a significant result, we found that there was no significant difference in *R^2^* values between dysplastic naevi and melanoma. However, the Wilcoxon Rank Sum test demonstrated significant differences in *R^2^* values between benign naevi and both dysplastic naevi and melanoma.

**Table 4:** Tests for differences in means between diagnostic groups with respect to fractal dimension and the regression coefficient of the lacunarity plot.

Inequalities	Test	T and z scores*	
		*Fractal dimension*	*Regression coeff.*
*Ben v Dys v Mel*	KW (*T*)	76.1 *p* = 0.0000	29.3 *p* = 0.0000
*Dys < Ben*	WRS (*z*)	3.9 *p* = 0.0001	4.8 *p* = 0.0000
*Mel < Ben*	WRS (*z*)	8.8 *p* = 0.0000	4.4 *p* = 0.0000
*Mel < Dys*	WRS (*z*)	4.4 *p* = 0.0000	−0.7 *p* = 0.4840
*Mel < Non-Mel*	WRS (*z*)	7.7 *p* = 0.0000	2.6 *p* = 0.0090

KW: Kruskal-Wallis.

WRS: Wilcoxon rank sum.

*Ben*: Benign.

*Dys*: Dysplastic.

*Mel*: Melanoma.

*Non-Mel*: Non-Melanoma.

All calculations are for intermediate maximal box size.

Positive *z* values reflect significance for the diagnostic group inequalities as shown. *p*-values are given to 4 significant digits.

* Results are for the red spectrum.

**Figure 4:**
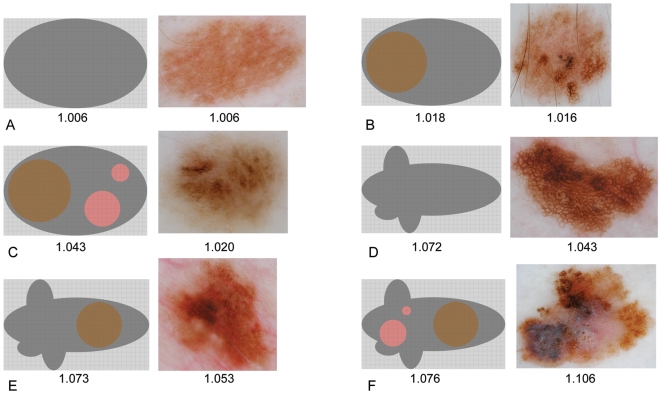
Schematic representation of pigmented lesion irregularity, associated examples, and their monotonically increasing lacunarity values. For the schematic representations, lacunarity was calculated using normalised red intensity obtained from the respective RGB colour space image. No irregularity (A), simple internal asymmetry (B), hierarchical internal asymmetry (C), boundary asymmetry (D), boundary asymmetry and simple internal asymmetry (E), and boundary asymmetry and hierarchical internal asymmetry (F).

Lacunarity analysis suggests there may exist an ordering of irregularity based on an images' singular lacunarity value. This classification is presented in Table 5 and illustrated schematically and by example in Fig. 4. To test whether the clinical implementation of this scheme correlates with calculated lacunarity values, two dermatologists expert in dermoscopy (HPS, JM) evaluated all 309 lesions at analysis resolution without prior knowledge of the diagnosis and assigned an irregularity score to each image in accord with this classification. In both cases Spearman's rank correlation was found to be positive (*r_s_* = 0.59, *p*<0.0001 and *r_s_* = 0.47, *p*<0.0001 respectively) thus demonstrating a statistically significant – although moderate – correlation between the clinically determined irregularity scores and their associated lacunarity values. The inter-observer agreement was found to be fair: the linear weighted kappa statistic between HPS and JM was 0.32.

**Table 5:** Melanocytic lesion irregularity classification.

1. Regular
* Lesion symmetry, smooth contour, well-defined edge*
* Homogeneous internal structure*
2. Simple internal asymmetry
* Lesion symmetry, smooth contour, well-defined edge*
* One or more internal structures of the same size and colour*
3. Hierarchical internal asymmetry
* Lesion symmetry, smooth contour, well-defined edge*
* Internal structures exhibiting two or more sizes and/or colours*
4. Boundary asymmetry
* Lesion asymmetry or scalloped contour or poorly-defined edge*
* Homogeneous internal structure*
5. Boundary asymmetry and simple internal asymmetry
* Lesion asymmetry or scalloped contour or poorly-defined edge*
* One or more internal structures of the same size and colour*
6. Boundary asymmetry and hierarchical internal asymmetry
* Lesion asymmetry or scalloped contour or poorly-defined edge*
* Internal structures exhibiting two or more sizes and/or colours*

**Figure 5:**
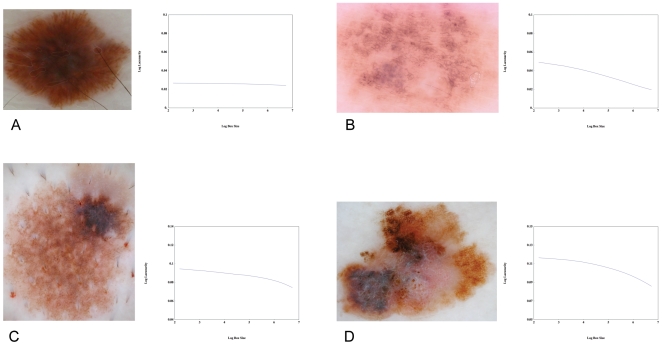
Lacunarity plots. The utility of lacunarity plots in yielding information about the geometric structure of an image is illustrated by considering the dysplastic naevi (A), (B), (C) and melanoma (D). Lesion A is uniform throughout without any discernable internal heterogeneity hence its corresponding lacunarity curve is straight and flat, indicating a scale-free structure and a fractal dimension very close to 2. Although the lacunarity curve of lesion B is straight, indicating a scale-free structure, the curve slopes downward, indicating a fractal dimension that deviates from 2. Its structure, over the length scale of the plot, is thus fractal. The plot of the dysplastic naevus (C) exhibits a downward inflection where the *x*-axis equals 5.5, revealing the existence of a prominent single-scale structural element of size ∼*e* ^5.5.^ = 16 *x*16 pixels. The melanoma shown in D exhibits significant heterogeneity of structure over many length scales, from the presence of small dark and pale regions to the prominent overall asymmetry. In contrast to A and B, its associated lacunarity curve is not straight because the image is not self-similar, while in contrast to C, a multi-scaled structure is revealed by the prominent curvature and absence of an inflection point. (All plots span the same range of log-lacunarity values, but not necessarily the same values).

Finally, lacunarity plots were generated for a sample cohort of images. We found that these plots can reveal additional information regarding the geometric structure of melanocytic lesions not accessible by the single-number lacunarity measure. They can be used to identify the typical length scale of irregularity in simple internal asymmetry (see Table 5), and can classify hierarchical internal asymmetry into either multi-scaled or fractal subtypes (Fig. 5).

## Discussion

In summary, our results demonstrate that lacunarity analysis is a potentially useful method of automating the assessment of melanocytic lesions. We now briefly explore its utility in the context of existing diagnostic algorithms.

The majority of investigators with an interest in the automated diagnosis of dermoscopic images of melanocytic lesions apply an artificial neural network [15], [16], [20]–[26] or a related algorithm [17], [27]–[29] to a training set of melanocytic lesions. For example, the aim of an artificial neural-network is to learn how to recognise various patterns of distinct inputs across multiple logic gates and respond with an appropriate output pattern. For melanocytic lesion diagnosis, the neural-network is usually one logic gate, and the inputs represent parameters in image evaluation. The network is set the task of optimising the number of inputs and their respective weights so that the output is appropriate to the given image [20]. The end result is a much smaller set of predictive variables, which are then used to test a sample cohort of images.

The range of sensitivities and specificities reported testify, in the main, to the effectiveness of the various automated procedures. However, there are a number of easily identifiable problems with their associated algorithms, some of which are outlined below. First, it is impossible to combine training-set results generated by different algorithms. The final sets of discriminatory parameters are different for each protocol, and the algorithms used in their implementation differ substantially. There exists an obvious imperative to reduce and standardise parameter sets. Second, there is a trade-off between the number of parameters used to discriminate melanocytic lesions and the ability of the algorithm to generalise accurately when applied to new lesions. High specificities flag the possibility of over-training with a consequent reduction in sensitivity when tested on new lesions, particularly in the differentiation of dysplastic naevi from early melanoma. Third, it is unclear whether images should be ‘cleaned up’ prior to analysis. Automated removal of artefacts such as hair shafts or air bubbles requires sophisticated image processing software and may create new artefacts. Fourth, identification of the boundary of a given lesion is problematic for computer algorithms, especially in melanoma or otherwise atypical naevi where regression and poorly defined borders are common. In one algorithm the investigator was required to manually draw the boundary in 24% of cases [30], rendering the process less than perfectly objective. Finally, many programs are licensed ‘black boxes’ hence their algorithmic details will never be completely transparent and open to independent scrutiny.

Lacunarity analysis has a number of inherent advantages over existing algorithms. First, as a measure with considerable overlap with previously reported diagnostic parameters, implementing the lacunarity calculation may facilitate a reduction in parameter numbers, thus reducing the risk of over-training [18]. Second, lacunarity analysis is algorithmically simple, transparent and fully reproducible. Third, the lacunarity algorithm is relatively insensitive to the presence of artefacts, such as hair shafts, that only occupy a small region of the image. Fourth, the results are robust to reductions in image resolution. The images analysed here were about 120 pixels wide; files of this size are easily electronically transferred by mobile phone where bandwidth limitations may exist. Fourth, our lacunarity analysis does not require the shape of the melanocytic lesion to be identified. Fifth, our lacunarity algorithm is readily applicable to non-polarised images since the major dermoscopic features and patterns found in polarised images are also present in non-polarised images [31]. Finally, the algorithm is readily applicable to multi-spectral images [32], with the caveat that discriminatory lacunarity values may need to be determined using a training set for each wavelength. It is possible that analysing images with our algorithm using specific visible or infrared wavelengths may be superior to broadband visible red.

Based on a pilot study of 309 dermoscopic images of melanocytic lesions, the lacunarity measure that characterises a given image has been shown to be a promising parameter in the automated differentiation of melanoma from non-melanoma. Lacunarity analysis can distinguish melanoma from benign naevi with a SE of 92 and a SP of 81, and it can distinguish melanoma from non-melanoma with a SE of 91 and a SP of 61. These figures are comparable to those previously reported for artificial neural networks and related diagnostic algorithms [15]–[17], [21], [25], [26].

Although the results presented above are encouraging, it is important to consider why our algorithm may fail to diagnose melanoma, why it may diagnose non-melanoma as melanoma, and whether it offers any particular advantages in difficult lesions. First, we consider the 9 cases where, with respect to the optimal lacunarity value used in the discrimination between melanoma and non-melanoma, the algorithm incorrectly diagnosed melanoma as non-melanoma. Surprisingly, 6 of those cases exhibited markedly diminished greyscale red intensity, thus accounting for their lower lacunarity values. We next consider cases where, with respect to the optimal lacunarity value used in their discrimination, dysplastic naevi were incorrectly diagnosed as melanoma. Examination of the greyscale red intensities for 14 of the dysplastic naevi associated with the highest lacunarity values, and diagnosed as melanoma, revealed a near absence of red intensity in the skin surrounding the lesions. This feature greatly increased contrast between the images and their surrounding skin, and led to images associated with high lacunarity values. We believe this is an artefact of the colour cast found in the clinical images. These anomalies in the degree of red intensity highlight the importance of standardising white balance, and suggest that a potentially important addition to the algorithm is to measure the red intensity throughout the lesion and its surrounding skin prior to analysis; if the maximum value is below some threshold then it may be better to calculate lacunarity for that image using another colour. Finally, we examined two melanomas where the clinical suspicion was moderate. In these cases, the lacunarity algorithm is able to identify pattern irregularity and textural changes that are not discernable with dermoscopic images.

Since heterogeneity of structure is one of the key dermoscopic features used to diagnose melanoma [33], the results presented herein confirm the hypothesis that as a quantitative measure of departures from translational invariance, lacunarity provides an objective assessment of melanocytic lesion irregularity. The lacunarity measure suggests a classification of lesion irregularity based on a monotonically increasing lacunarity value, and we are encouraged by our preliminary results that suggest it may be possible for the ordering of this classification to be determined clinically. We expect observer performance (including inter-observer agreement measures) will improve with further training. The utility of this scheme in the clinical dermoscopic evaluation of melanocytic lesions is currently unknown but its implementation may improve clinical diagnostic accuracy and therefore warrants further investigation.

It is well known that the boundaries of malignant lesions may exhibit fractal-like geometries. The fractal dimensions of melanocytic lesion border [34] or surface [18] have been previously investigated, demonstrating increasing boundary fractal dimension with lesion progression in the former, and no difference between diagnostic groups in the latter. Here we have found, in contrast to the results presented by Manousaki *et al* above, that the two-dimensional image fractal dimension is near 2 for benign naevi, lower for dysplastic naevi, and lower again for melanoma. It is not surprising that benign naevi are associated with a fractal dimension close to 2 since, in general, their two-dimensional dermoscopic images fill space uniformly. However, it is important to note that any object can be assigned a fractal dimension, irrespective of whether it actually *is* fractal. A fractal object is defined as one that is self-similar across all length scales, and is characterised by a linear lacunarity plot. If the lacunarity plot is not linear, then assigning a single value (the fractal dimension) to the slope of the line may not be meaningful. We found that the degree of self-similarity (Pearson's correlation for the line of best fit to the lacunarity plot), could not distinguish dysplastic naevi from melanoma, hence we conclude that melanoma is not more ‘fractal-like’ than dysplastic naevi, although we have found melanoma may exhibit a lower fractal dimension. Although we found benign naevi were more fractal-like than either dysplastic naevi or melanoma, this is an artefact since their self-similarity is simply due to their uniformity. Finally, although we have found that irregular melanocytic lesions with fractal dimensions that are less than 2 are either true fractals (Fig. 5b) or multi-scaled (possibly multi-fractal, Fig. 5d), it is an open and interesting question as to whether this distinction has any biological significance.

In conclusion, we have demonstrated the utility of lacunarity analysis in assessing the patterns and textures found in melanocytic lesions. We suggest lacunarity analysis may find utility in melanocytic lesion assessment as either part of an artificial neural-network reduced parameter set or as a stand-alone measure. Although this pilot study suggests it has diagnostic potential in the automated classification of melanoma from non-melanoma, prospective studies are required for validation. Lacunarity analysis suggests there may exist an ordering of irregularity in melanocytic lesions, and further investigation is required to determine whether this ordering has any clinical utility.
